# Asexual reproduction and vegetative growth of *Bionectria ochroleuca* in response to temperature and photoperiod

**DOI:** 10.1002/ece3.7856

**Published:** 2021-06-29

**Authors:** Yi Zheng, Yichun Xie, Yan Xie, Shixiao Yu

**Affiliations:** ^1^ Department of Ecology/School of Life Sciences State Key Laboratory of Biocontrol Sun Yat‐sen University Guangzhou China; ^2^ School of Life Sciences The Chinese University of Hong Kong Shatin, New Territories Hong Kong SAR China

**Keywords:** conidiation, environmental factors, growth, reproduction, trade‐off

## Abstract

Growth and reproduction are two essential life‐history traits for fungi. Understanding life‐history strategies provides insight into the environmental adaption of species. Here, we investigated the colonial morphology, vegetative growth, and asexual reproduction of the ascomycete fungus *Bionectria ochroleuca* in response to a variety of environmental conditions. We demonstrated that the increased temperature from 15 to 25°C induced mycelial growth and conidiation in *B. ochroleuca*. We also found that the optimal temperatures for mycelial growth and conidial formation in this fungus species were 25 and 30°C, respectively. However, as the temperature increased from 25 to 30°C, mycelial growth was suppressed, but the total number of conidia was significantly increased. The shift in light–dark cycles dramatically changed the morphological features of the colonies and affected both vegetative growth and asexual reproduction. Under incubation environments of alternating light and dark (16:8 and 8:16 light:dark cycles), conidiophores and conidia in the colonies formed dense‐sparse rings and displayed synchronous wave structures. When the light duration was prolonged in the sequence of 0, 8, 16, and 24 hr per day, mycelial growth was suppressed, but conidiation was promoted. Together, our results indicate that temperature and light period may trigger a trade‐off between vegetative growth and asexual reproduction in *B. ochroleuca*.

## Graphical abstract

The trade‐offs between vegetative growth and reproductive growth are essential to the environmental adaptation and population growth of the species. However, scientists have not found the evidence in support of either a positive or negative relationship between growth and reproduction. By using *Bionectria ochroleuca*, a potential pathogen which was isolated and purified from a subtropical tree *Cyclobalanopsis chungii*, we investigate its changes and differences in vegetative growth and reproductive growth under a variety of environmental conditions. Our results demonstrated that the compensation of limiting resources would induce both reproduction and growth, and the high plasticity on light, nutrient, and temperature conditions during conidia germination is the adaptive advantages for survival and dispersal in changeable environments.

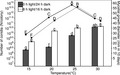

## 1 INTRODUCTION

Phenotypic plasticity is the ability of a given genotype to produce different phenotypes in various environments (Holloway, 2002) and is a fundamental characteristic of all organisms, enabling adaptation to various environments (Slepecky & Starmer, 2009). The molecular basis of phenotypic plasticity is differential gene expression triggered by environmental changes (Alvarez et al., 2015; Gibson, 2008). Such regulation includes early signal responses, signal transduction, and gene expression regulation (Lengeler et al., 2000). Gene expression is regulated at various stages, including epigenetic (Felsenfeld & Groudine, 2003), transcriptional (Levine & Tjian, 2003; Tuch et al., 2008), and post‐transcriptional regulations (He & Hannon, 2004; Palazzo & Lee, 2015). These regulations are quite capable of changing growth forms (Rayner & Coates, 1987), colonial morphology (Bago et al., 2004), and physiological states (Alberton et al., 2005) in response to environmental stimulation.

Visible light is an important signal that can be perceived by organisms. Light signaling regulates metabolic pathways in fungi (Kamada et al., 2010; Tisch & Schmoll, 2010). Visible light can trigger a molecular pathway, generating an oxidative stress response, which leads to repressed mycelial growth of some fungal species, and it also plays a role as the signal of more harmful ultraviolet (UV) light (Fuller et al., 2015). Previous studies reported that light could repress the mycelial growth of numerous fungi, such as *Botrytis cinerea* (Canessa et al., 2013), *Aspergillus fumigatus* (Fuller et al., 2013), and *Cordyceps militaris* (Yang & Dong, 2014), but its effect on conidiation was not consistent across different fungi. White light promoted conidiation via the WC‐1 ortholog, LreA, and phytochrome FphA in *Aspergillus nidulans* (Bayram et al., 2010), while it repressed conidiation in *Aspergillus oryzae* (Hatakeyama et al., 2007) and *Aspergillus alternata* (Pruß et al., 2014).

Environmental temperature is a ubiquitous environmental stress affecting fungal growth (Li et al., 2009). Temperature can dramatically affect spore germination (Ayerst, 1969; Plaza et al., 2003; Yeo et al., 2003), mycelial growth (Li et al., 2009), reproduction (Brasier, 1969), and colony morphology (Li & Nielsen, 2017). For pathogenic fungi, temperature and its related signaling cascades regulate morphogenesis, which directly affects virulence factors of the pathogen (Bernard et al., 2013; O'Meara & Cowen, 2014).

Vegetative growth and reproduction are two vital life‐history traits and important components of fitness (Stearns, 1989). These two processes are internally connected by physiological factors. In addition, physiological and environmental factors together drive the life‐history strategy (Anderson et al., 2019; Kües, 2000). How the fungus responds to the changing environment with different life strategies is key to understanding developmental fitness and adaptation. Reproduction is an energetically intensive process. It requires energy and materials that are acquired and stored during vegetative growth (Harshman & Zera, 2007). In fungi, the initiation of sexual reproduction is related to the accumulation of glycogen, a common storage compound, in mycelia during vegetative growth (Badaruddin et al., 2013; Brunt & Moore, 1989). However, the energy and resources acquired by organisms are limited. Therefore, a balance, or more specifically, a negative relationship between vegetative growth and reproduction is expected. In response to different nutritional environments, *Neurospora crassa* displayed a highly dynamic balance between asexual and sexual development, during the switch from vegetative growth to reproduction (Wang et al., 2019). In *Coprinopsis cinerea*, major differences in carbohydrate metabolism and carbohydrate redistribution were observed during fungal development, from vegetative growth to conidiation, fruiting, and sclerotia formation under different environmental conditions (Xie et al., 2020). Such balancing processes were internally regulated by regulatory genes and metabolic genes with high flexibility (Wang et al., 2019; Xie et al., 2020).

*Bionectria ochroleuca* is a fungus that has been isolated worldwide from the soil (Schroers et al., 1999) and plant tissues, such as roots, stems, and leaves (Ebrahim et al., 2012; Paul et al., 2013; Zakaria et al., 2016). It has both sexual and asexual phases, and perithecia and conidiophores are formed in each phase, respectively (Schroers et al., 1999). It has been identified as a plant pathogen of several agrospecies (Bienapfl et al., 2012; Guesmi‐Jouini et al., 2014; Holguín‐Peña et al., 2012; Li et al., 2014), an endophyte (Samaga et al., 2014), or a free‐living form (Kim et al., 2019). The change between these lifestyles is strongly influenced by environmental factors and plant defense responses (Schulz & Boyle, 2005). Case studies showed that direct spore spray to leaves can cause fungal colonization that was later found in the whole aerial part of artichoke (*Cynara scolymus*) (Guesmi‐Jouini et al., 2014). It can also be transferred from the soil to roots, causing root rot and systemic colonization in soybeans (Bienapfl et al., 2012). In this study, we conducted control experiments on a *B. ochroleuca* isolate to investigate the impacts of environmental factors on conidiation, conidial germination, and vegetative growth of the fungus. Specifically, we determined the (a) colonial morphology of the fungus under different temperatures and light conditions; (b) optimal temperature and light duration for asexual reproduction and vegetative growth; and (c) relationship between reproduction and vegetative growth under different environmental conditions.

## 2 MATERIALS AND METHODS

### 2.1 Field environment and fungal isolation

The fungi used in this study were isolated from damping‐off seedlings of *Cyclobalanopsis chungii* in a subtropical forest in the Heishiding Nature Reserve (Guangdong Province, China; 111°53′E, 23°27′N). This forest is in a subtropical monsoon climate zone. The mean annual temperature is 19.6°C, and the mean monthly temperatures range from 10.6°C in January to 28.4°C in July, according to Liu et al. (2016). Day length is approximately 14 hr in the summer and 10 hr in the winter.

The fungal strains were isolated from damping‐off *C. chungii* seedlings following the protocol described by Cao et al. (2004) with modifications, as follows: (a) root segments were washed with autoclaved water three times and five times before and after surface sterilization, respectively; and (b) the roots were treated with 10% sodium hypochlorite for 2 min. After surface sterilization, the root segments were placed onto potato dextrose agar (20 g potato starch, 4 g dextrose, and 15 g agar in 1 L) and incubated at 25°C under an 8‐hr light/16‐hr dark regime for 10 days. Emerged fungal isolates were purified by the streaking plate method to separate co‐isolated strains. A total of 60 strains were isolated. Pure cultures of the strains were kept on a PDA slant at 4°C as the short‐term stock.

### 2.2 Identification of *B. ochroleuca* isolates

One of the 60 isolates, Cch11, generated concentric circle‐like colonies under stationary light–dark regimes on PDA plates, which stimulated our interest. Genomic DNA of the strain was extracted from fresh mycelia grown on PDA using a DNeasy Plant Mini Kit (Qiagen). The internally transcribed spacer (ITS) region was amplified using ITS1 (5′‐TCCGTAGGTGAACCTGCG‐3′) and ITS4 (5′‐TCCTCCGCTTATTGATATG‐3′) primers (White et al., 1990) with the KAPA HiFi HotStart ReadyMix PCR kit (Roche) under the following program: 95°C for 3 min, followed by 30 cycles of 98°C for 20 s, 65°C for 20 s, and 72°C for 15 s, and 72°C for 1 min. PCR products were detected on 1.5% agarose gel and were purified with the MEGA quick‐spin Plus Fragment DNA Purification Kit (MEGA). Sanger sequencing of PCR products was performed on a 3,730 xl DNA Analyser (Applied Biosystems).

To double confirm that the pure culture contained only Cch11, spores from the culture were collected and plated. We further randomly selected 20 colonies from the plates and performed PCR amplification and Sanger sequencing. We used R package “ape” v5.4‐1 (Paradis & Schliep, 2019) to construct the phylogenetic tree of Cch11 and the targeted fungal species from GenBank (Table S1). The distance was estimated with Kimura's 2‐parameter distance model (Kimura, 1980) using the “dist.dna()” function. A phylogenetic tree was constructed using neighbor‐joining method (Saitou & Nei, 1987) using the “nj()” function. Assessment of the statistical confidence of the inferred relationships was performed by 1,000 bootstrap replicates using the “boot.phylo()” function (Felsenstein, 1985). The tree was visualized using R package “ggtree” v2.2.4 (Yu et al., 2017).

### 2.3 Effect of temperature and photoperiod on mycelial growth and asexual reproduction

The short‐term stock of *B. ochroleuca* at 4°C was activated, subcultured, and grown at 25°C on PDA medium for 10 days to obtain a working culture. To perform tests, the fungal conidia were transferred from the working culture and inoculated on the center of a plate with an inoculation needle. The newly inoculated plates were randomized before incubation and each treatment group contained 15 plates as replicates. To determine the effect of temperature on fungal growth, the plates were incubated at 15, 20, 25, and 30°C under continuous darkness or an 8‐hr light/16‐hr dark regime for 10 days. Considering that lighting conditions at the soil surface under the forest canopy are affected by terrain, canopy cover, and other factors, the duration of light exposure on the ground will be less than the total day length. The light duration was set to 8 hr per day, as one of the major experimental groups. In contrast, continuous dark mimicked the underground environment. To investigate the effect of photoperiod on fungal growth and reproduction, fungal cultures were incubated at 25°C with 0, 8, 16, and 24 hr light exposure per day for 10 days. The cultures were exposed to full light induced by fluorescent lamps with 800–1,200 lx. The light intensity of the experiment did not exceed the range in the field, where it was 200–800 lx in the understory and 800–2,500 lx on the southern slopes, according to our measurements. We did a supplementary experiment to confirm that the inoculation concentration did not have a strong influence on mycelial growth and conidiation (see the supplementary file for details). Mycelial growth was measured as the mean of two randomly selected orthogonal diameters of the colony on day 10 with a ruler. The colonies were in the shape of near‐perfect circles, with differences between two diameters <1%. The conidia were then washed out using a spreader with 10 ml ddH_2_O. A spore suspension was transferred to sample tubes with a pipette and quantified using a hemacytometer (XB.K.25, Shanghai Qiujing Biochemical Reagent and Instrument). Microscopic examination showed that almost no conidia were found on the culture after scratching and wash. ANOVA was performed using the “aov()” function in R 4.0.2 (R Core Team, 2020). Each ANOVA was followed by Tukey's honestly significant difference test to identify significant differences using the “TukeyHSD()” function. Multiple comparisons were done using the “HSD.test()” function in R package “agricolae” v1.3‐3 (de Mendiburu, 2017). Data were visualized using R package “ggplot2” v3.3.2 (Wickham, 2016).

### 2.4 Effect of different environmental conditions on conidial germination

To investigate the effect of nutrients, temperature, and light on conidial germination, freshly grown conidia were collected 5 days postinoculation from colonies grown on PDA. Conidia were washed off PDA with ddH_2_O using a spreader, and the spore suspension was adjusted to approximately 10^7^ spores/ml for the following tests. To test the impact of nutrient concentration on conidial germination, the conidia suspension was mixed with different concentrations of potato dextrose broth (PDB) to achieve a final nutrient concentration of 0%, 0.1%, 1%, 2%, and 5% and was then incubated at 25°C under continuous darkness. To determine the optimal temperature for conidial germination, conidia were incubated in 1% PDB at 4, 15, 20, 25, and 30°C under continuous darkness. In the light study, conidia were cultured in 1% PDB at 25°C under continuous light or darkness. All assays had a final spore concentration of 5 × 10^6^ conidia/ml. All the above incubation treatments were performed by placing 25 μl of the freshly mixed spore‐PDB/H_2_O suspension onto each slide and placing the slides in a Petri dish with a moistened filter paper (Odell & Smith, 1982). Conidial germination was examined under a light microscope (400×) 3, 6, 9, 12, 24, and 36 hr after incubation. These assays were performed on three technical replicates, and five fields were examined for each slide. When over 85% of the conidia in the observation field on a slide germinated, measurements on that slide were terminated. Photographs were adjusted and processed in Photoshop CS 6. A conidium was considered germinated when the germination tube exceeded one half of the largest dimension of the conidium (Gottlieb, 1978). ANOVA, multiple comparison, and data visualization were performed using R 4.0.2 (R Core Team, 2020), as described in previous sections. All controlled experiments, namely mycelial growth, conidia induction, and conidial germination, were repeated three times, and the results were consistent.

## 3 RESULTS

### 3.1 Identification of the isolated *B. ochroleuca* strain

The ITS region of strain Cch11 (see the blast result in the supplementary file) was successfully sequenced, and it was most similar to NCBI sequence ID HQ157202.1, a partial sequence belonging to *B. ochroleuca* isolate RZ, with 99% identity. All 20 colonies showed the same colony characteristics and the same ITS sequence, certifying their purification. The new isolate showed a very even distance with the endophytes, plant pathogens, and free‐living groups, according to the phylogenetic tree (Figure S1).

### 3.2 Morphology of the isolated *B. ochroleuca* strain

**FIGURE 1:**
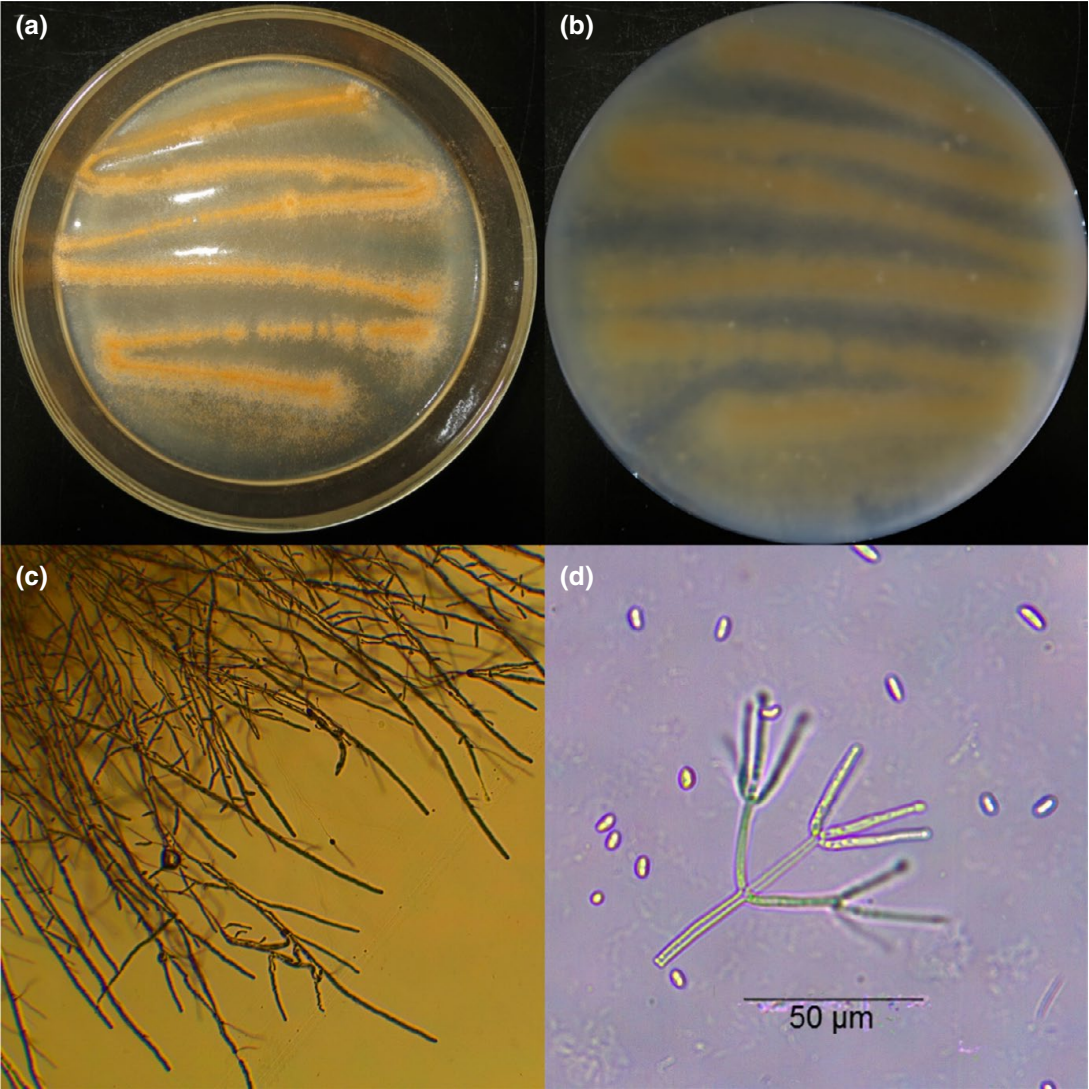
Isolation of *Bionectria ochroleuca* from *Cyclobalanopsis chungii*. (a) Colony surface and (b) reverse colony surface incubated at 25°C in an 8‐hr light/16‐hr dark regime. Microscopic features of (c) vegetative mycelia and (d) verticillium‐like conidiophores and conidia

**FIGURE 2:**
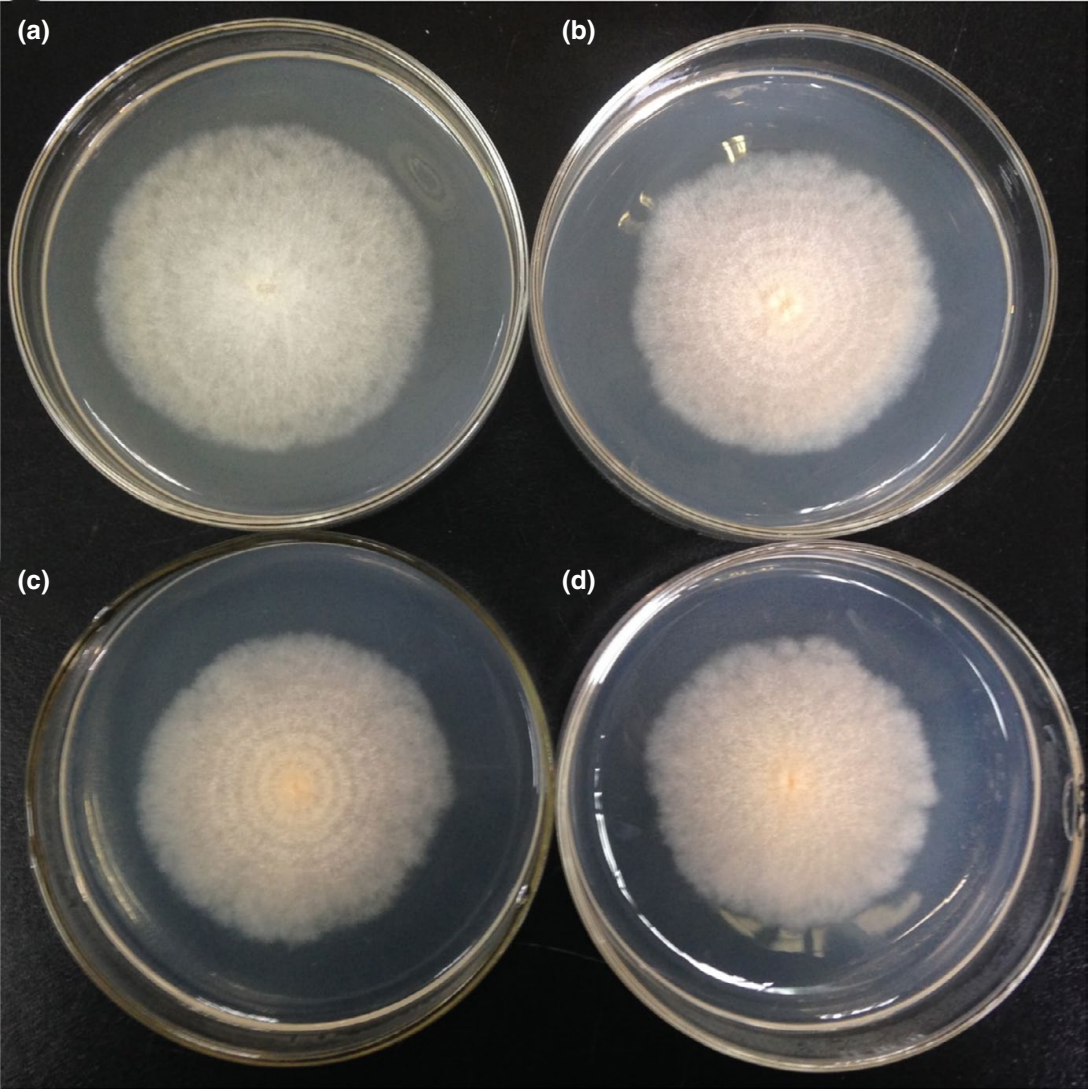
Surface of *Bionectria ochroleuca* colonies grown at 25°C under different light–dark regimes. (a) Continuous darkness; (b) 8‐hr light/16‐hr dark regime; (c) 16‐hr light/8‐hr dark regime; and (d) continuous light

The morphological features of *B. ochroleuca* Cch11 are shown in Figure 1. Mycelial growth at 25°C was 52.0–60.0 mm in colony diameter on day 10 and varied according to the duration of light. The fungal culture grown under light exposure was light orange with a considerable number of conidia on the surface (Figure 1a). The opposite side of the colony was a pale yellow to yellow color (Figure 1b). Colonies grown under continuous darkness were not pigmented and had a white appearance (Figure 2a). Colonies produced concentric rings under the stationary light–dark regimes (Figure 2b,c). The growth ring formed in the light period had a darker color, whereas the growth ring formed in the dark period had a paler color, and synchronous wave structures were observed (Figure 2b,c). In the dense area of the concentric ring, conidiophores and conidia were strongly clustered (Figure S2a), whereas in the sparse area, the size of the conidiophore cluster was smaller, and density was lower (Figure S2b). The intermediate line of dense and sparse areas was clear under a high‐power light microscope (400×, Figure S2c). The surfaces of colonies grown under continuous light or darkness were uniform and lacked a ring structure (Figure 2a,d). Clustered conidiophores and conidia were rarely found in colonies grown under continuous darkness. The conidia pellet had a white to pale pink color. We did not find sexual structures such as ascomata, asci, or ascospores in the microscope fields.

### 3.3 Light inhibited mycelial growth but promoted the conidiation of *B. ochroleuca*

**FIGURE 3:**
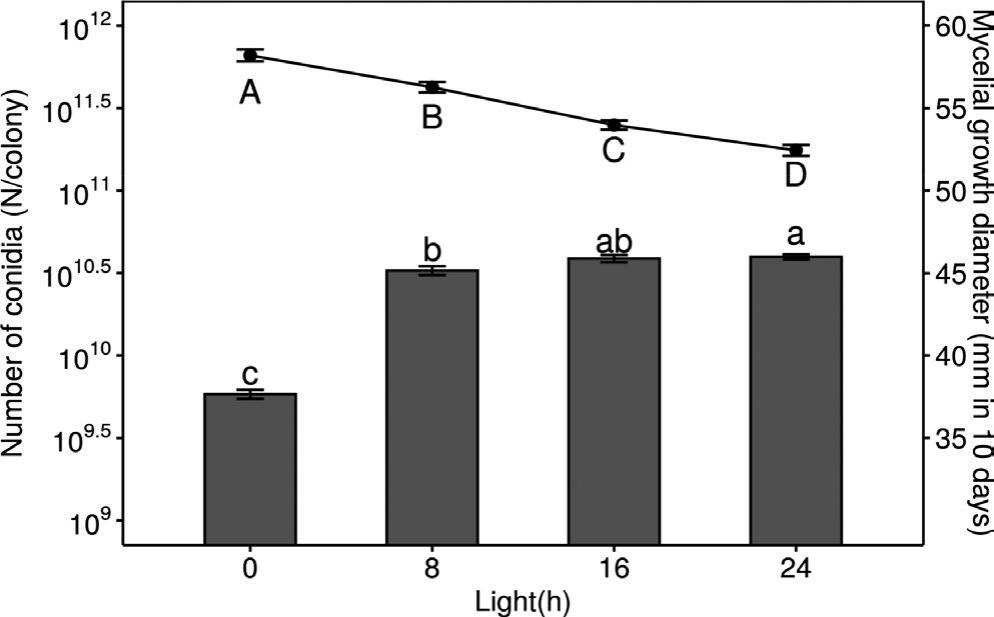
Hyphal growth and conidiation at 25°C under different light‐dark regimes. The bar plot shows the number of conidia after 10 days of incubation. The dot plot shows the mycelial growth diameter. Error bars show the standard error of 15 plates in each group. Lowercase and uppercase letters show conidia number and mycelial growth diameter, respectively, that are significantly different among groups (*p* < 0.05)

**FIGURE 4:**
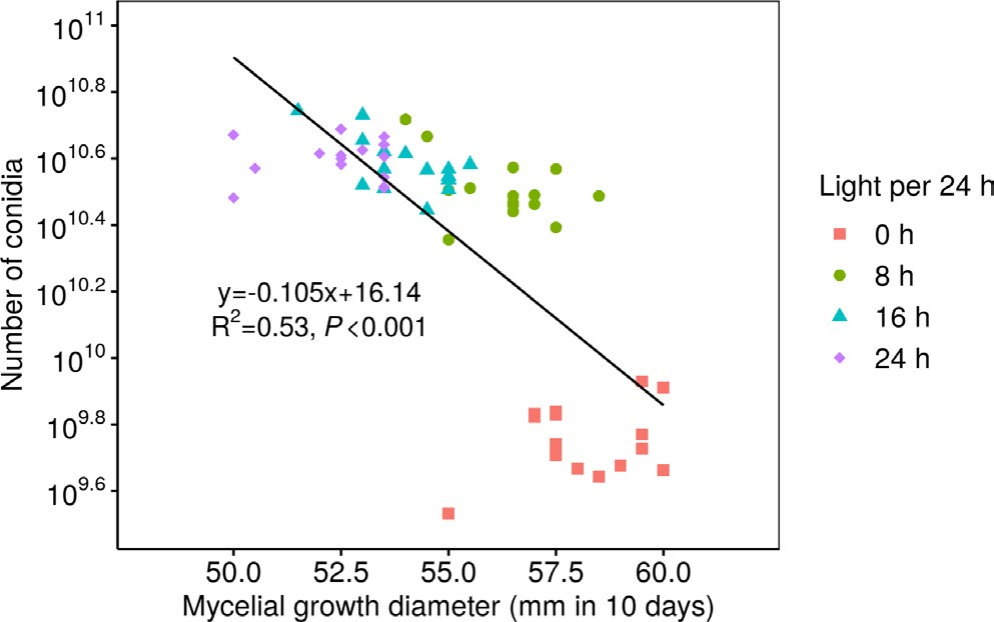
Trade‐off between conidiation and vegetative growth of *Bionectria ochroleuca* at 20°C. The symbols represent colonies incubated for 0 (red squares), 8 (green circles), 16 (blue triangles), and 24 hr (purple squares) of light per day

At 25°C, *B. ochroleuca* showed a highest mycelial growth of 58.0 ± 0.4 mm in colony diameter in 10 days under continuous darkness, and growth significantly decreased as the time of light exposure increased (Figure 3). A lowest growth of 52.0 ± 0.3 in 10 days was observed under continuous light. In contrast, the production of asexual spores, that is, conidiation, was strongly promoted by light (Figure 3). Without light, the fungus produced 5.83 ± 0.37 × 10^9^ conidia in 10 days. The number increased 5.6‐fold under the 8‐hr light/16‐hr dark regime treatment, and maximum conidia formation (3.97 ± 0.14 × 10^10^) conidia occurred under continuous light exposure. We observed a negative correlation between mycelial growth and the total number of conidia at 25°C (Figure 4), which indicated a light‐regulated trade‐off between vegetative growth and asexual reproduction in *B. ochroleuca*.

### 3.4 Mycelial growth was significantly different at different temperatures

**FIGURE 5:**
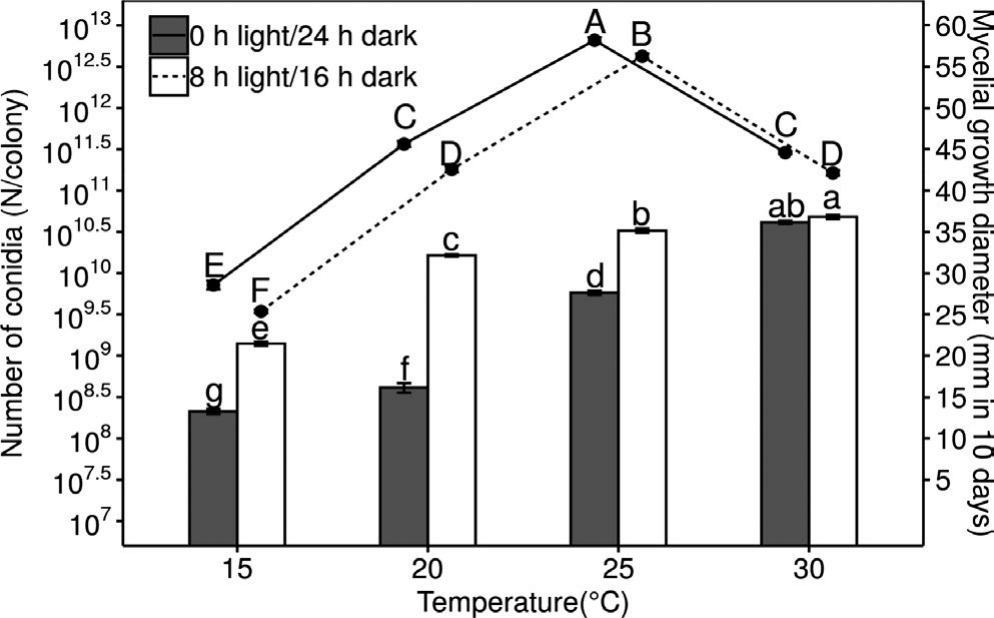
Mycelial growth and conidiation at different temperatures and photoperiods. The bar plots show the number of conidia after 10 days of incubation. Dark gray bars represent conidia production under continuous darkness, while white bars represent conidia production under an 8‐hr light/16‐hr dark regime. Dot plots show the mycelial growth (the colonial diameter on day 10), in which the solid line indicates the groups grown under continuous darkness, and the dotted line indicates the groups grown under an 8‐hr light/16‐hr dark regime. Error bars show the standard error of 15 plates in each group. Lowercase and uppercase letters show the conidia number and mycelial growth diameter, respectively, that are significantly different among groups (*p* < 0.05)

To determine the impact of different temperatures on *B. ochroleuca* development, cultures were incubated at a range of temperatures. *B. ochroleuca* achieved maximum mycelial growth at 25°C, and growth decreased by 25%–40% with every 5°C change in incubation temperature (Figure 5). Under different photoperiods, mycelial growth showed a similar pattern in response to temperature change, although it was inhibited by light, which caused a 10%–20% decrease in growth at all temperatures.

### 3.5 High temperatures overrode conidiation repression in the dark environment

Conidiation increased with incubation temperature under each of the two experimental photoperiod conditions (0‐hr light/day and 8‐hr light/day; Figure 5). At relatively lower temperatures of 15, 20, and 25°C, the fungus produced only 11.9%–16.5% of the conidia under continuous darkness compared to the light‐exposed cultures. In contrast, at a high temperature of 30°C, conidiation had no significant difference between the two photoperiod conditions.

### 3.6 External nutrient supply promoted conidial germination

Under incubation at 25°C, few conidia germinated within the first 3 hr, regardless of the nutrient conditions, indicating a conidia activation duration of 3 hr ( Figure S3). The external nutrient supply of 1% or a higher concentration of PDB significantly promoted conidial germination. Isotropic growth of conidia was observed between 3 and 9 hr after incubation, and germination tubes were mainly formed between 6 and 12 hr. In 1% PDB, conidia displayed a germination rate of 93.3 ± 0.3% at 24 hr, and in 5% PDB, the germination rate further increased and exceeded 95%. Conidial germination was relatively slower in 0.1% PDB and H_2_O. However, approximately 80% of the conidia germinated in 0.1% PDB and in clear water after 24 hr of incubation. These results proved that an external nutrient supply promotes but is not necessary for *B. ochroleuca* conidial germination.

### 3.7 High temperatures accelerated conidial germination by shortening the activation period

**FIGURE 6:**
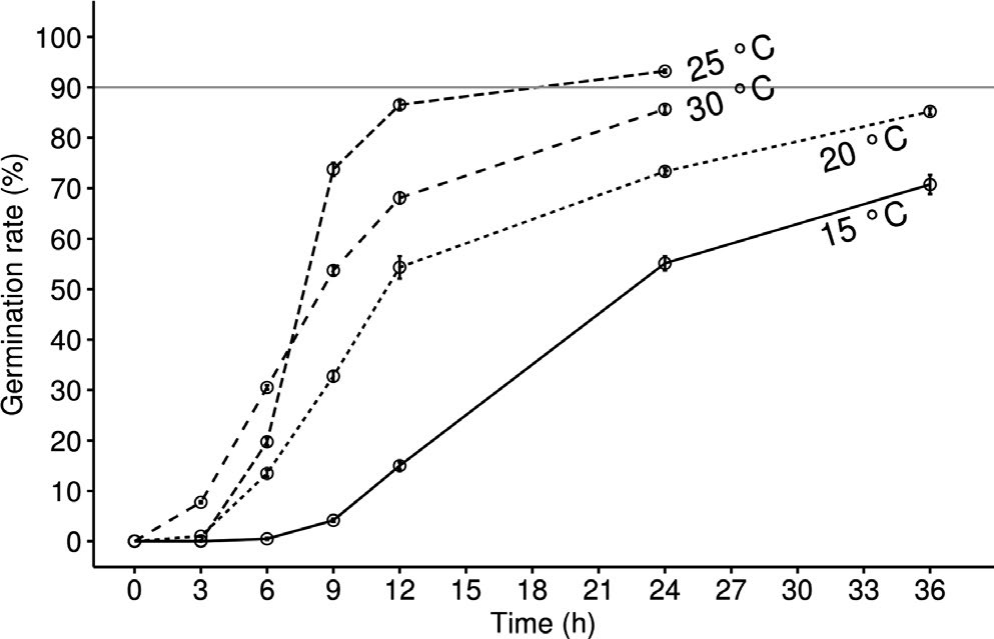
Conidial germination rate under different temperatures. Error bars show the standard error of 15 observation fields

When conidia were incubated at different temperatures, the germination speed and rate varied (Figure 6). At 30°C, approximately 10 and 30% of the conidia germinated after 3 and 6 hr of incubation, respectively; these values were double those observed at 25°C. However, only 85% of conidia germinated after 24 hr of incubation at 30°C, which was slightly lower than the germination rate at 25°C (> 90%). This showed that the germination activation period was significantly shorter at 30°C, but the germination rate of conidia was slightly decreased. At 15°C, the duration of germination activation was extended and estimated to be between 6 and 9 hr. Fewer than 75% of the conidia germinated after 36 hr of incubation. Although a high temperature of 30°C shortened the conidia activation time, 25°C was the optimal temperature for *B. ochroleuca* to achieve the highest germination rate in a reasonable time duration.

### 3.8 Light exposure did not have strong effects on conidial germination

**FIGURE 7:**
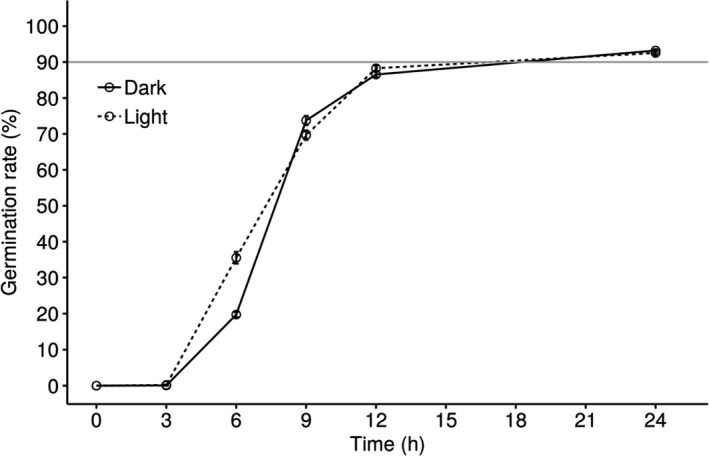
Conidial germination rate under different light conditions. The solid line represents continuous darkness, while the dashed line represents full‐time light exposure. Error bars show the standard error of 15 observations

After 3 hr of incubation, no significant difference was detected between conidial germination in continuous darkness and full‐time light exposure (Figure 7). The germination rate was higher under full‐time light exposure after 6 hr of incubation, but the results were reversed after 9 hr of incubation (Figure 7). When conidia were incubated for 24 hr, the germination rate in both conditions exceeded 90% and were not significantly different (Figure 7).

## 4 DISCUSSION

Flexible allocation between vegetative growth and reproduction is essential for the environmental adaptation of a species. In this study, we monitored the colonial morphology, mycelial growth, conidiation, and conidial germination processes of *B. ochroleuca* under different environmental conditions. We found light‐induced pigmentation, and the colonies formed concentric dense‐sparse rings under the stationary light–dark regimes. Mycelial growth and conidial germination showed a unimodal‐type response to the increase in temperature, while conidiation was promoted constantly. Mycelial growth was suppressed, but conidiation was promoted with the increase in light duration. This is the first report of trade‐offs between vegetative growth and asexual reproduction in this fungus. Sexual reproduction was not detected during the experiment.

### 4.1 Regulation of growth morphology improves fitness in a changing environment

Light is a critical environmental factor for fungal development (Bahn et al., 2007; Sakamoto, 2018). It works as a stimulus for fungi and can indicate time, space, stress, and disturbance (Fuller et al., 2015; Rodriguez‐Romero et al., 2010). In response to light, fungi undergo nonmorphological changes or metabolic changes, including the regulation of carotenoid and carbohydrate metabolism, as well as morphological changes in sporulation and sporophore formation (Tisch & Schmoll, 2010).

In this study, light‐induced pigmentation was observed in *B. ochroleuca* cultures. Previous studies documented that the production of the orange pigment in *B. ochroleuca* strongly depended on light and was formed particularly under UV light (Schroers et al., 1999). Pigment accumulation can protect fungi against UV rays (Mukherjee et al., 2017). Although the fungal strain in this study was isolated from rotten seeds and regarded as a soil‐borne pathogen, this species was previously reported to be able to infect strawberry leaves as an endophyte (Costa et al., 2013). Therefore, pigmentation in response to light might protect it from potential light stress. In addition, the regulation of pigment biogenesis, as well as other secondary metabolites, also helps fungi to survive under other types of environmental stress, including extreme heat/cold and drought (Treseder & Lennon, 2015).

Fungi occupy new habitats via the growth of mycelia and the release of spores and other reproductive units. We found that both of these processes were regulated by light in *B. ochroleuca*. The presence of light conveys the signal of an open area, which is suitable for fungal dispersal, whereas a lack of light indicates a hermetic space (Rodriguez‐Romero et al., 2010). As light duration increases, the growth of mycelia is repressed and conidiation is significantly promoted. In contrast, in a dark environment, fungi develop more aerial hyphae but fewer conidia because connected hyphae are a more appropriate way than conidia to expand the habitat belowground (Fuller et al., 2015; Halbwachs et al., 2016; Lee et al., 2006). As the change from vegetative growth to asexual reproduction can be energetically and materially costly, such light‐induced responses have selective advantages for fungal species (Fuller et al., 2015). We did not find characteristic structures of sexual phase during the experiment. Relevant studies reported that perithecia, a special type of ascomata of *B. ochroleuca*, were generally found on natural substratum such as bark (Schroers et al., 1999).

### 4.2 Conidial germination of *B. ochroleuca* adapts to various environmental conditions

Spore germination is one of the essential steps in the fungal life cycle and affects the survival and dispersal abilities of fungi (D'Enfert, 1997). It has been proven in a range of wood‐decaying basidiomycetes that sensitivity to sunlight and freezing restricts the dispersal of fungi (Norros et al., 2015). Our results showed that the conidial germination of *B. ochroleuca* was sensitive to temperature. An increase in temperature shortened the time of activation during germination, especially at 30°C, at which mycelial growth was repressed, but significantly faster conidial germination was observed. Such a phenomenon indicated that the germination of *B. ochroleuca* conidia can be activated by moderate heat, similar to the heat‐activating effects on the germination of sexual and asexual spores in other ascomycetes and basidiomycetes (Dunkle, 1975; Feofilova et al., 2004; Gottlieb, 1950). In addition, in *N*. *crassa*, *Aspergillus niger*, and other species, it has been demonstrated that nutrients from the environment were necessary for conidial germination (Abdel‐Rahim & Arbab, 1985; Hayer et al., 2014; Wang et al., 2019). However, our assays showed that an external nutrient supply promoted but was not necessary for conidial germination of *B. ochroleuca*. Under all experimental conditions used in this study, including light, temperature, and nutrient gradients, *B. ochroleuca* conidia germinated within 2 days, with high germination rates of 75%–95%. As the fungus can be endophytic, pathogenic, and free‐living under different environmental conditions (Samaga et al., 2014), we presume that the high plasticity of conidial germination in response to nutrient and temperature has adaptive advantages for survival and dispersal in changing environments.

### 4.3 Environmental factors affect the trade‐off between vegetative growth and asexual reproduction

The mycelial growth and conidiation of *B. ochroleuca* both increased as inoculation temperature increased from 15 to 25°C. As temperature further increased to 30°C, mycelial growth was decreased, indicating a high temperature suppression effect. Conidiation, in contrast, did not appear to reach the upper limit. These results suggest a life‐history strategy of shifting energy allocation from vegetative growth to reproduction in response to stress (Stearns, 1989). Previous studies also found a similar allocation strategy in other plant pathogens under stressful environments, including in *Fusarium solani*, *Neoscytalidium hyalinum*, and *B. cinerea* (Boumaaza et al., 2015; Hohenfeld et al., 2018).

The fungus *B. ochroleuca* traded vegetative growth for higher conidia production under a prolonged photoperiod. Photoperiod is an indicator of time, which affects the biological clock. A previous study found that the biological clock could control the conidia‐releasing process of fungi (Yoshida et al., 2008). The effects of light on sporulation are quite varied among different fungal species. For several fungal species, sporulation required both light and dark periods (Griffin, 1996). However, the prolonged photoperiod contentiously increased conidia production of *B. ochroleuca*, and the fungus could produce conidia under all photoperiod conditions (0, 8, 16, and 24 hr/day). This indicated that light was neither a necessary requirement nor an inhibitor for sporulation of *B. ochroleuca*. The extension of light duration significantly reduced mycelial growth of the fungus. Previous studies found that visible light itself could be a source of oxidative stress (Rangel et al., 2015). In *B. cinerea,* it led to a reduction in the growth rate, but such repression could be rescued simply by the addition of the antioxidant ascorbate to the medium (Canessa et al., 2013). In general, however, the major effects of light on fungi are on sporulation rather than on growth (Griffin, 1996). For example, increasing the photoperiod slightly increased conidia production, but had no significant influence on the mycelial growth of *Valdensinia heterodoxa* (Vogelgsang & Shamoun, 2002). Similar patterns were found among other fungal species (Alves et al., 2009; Avery et al., 2004). However, little evidence for the controversial effects of photoperiod on growth and sporulation was found in previous studies. As far as we know, this is the first evidence for trade‐offs between mycelial growth and asexual reproduction in *B. ochroleuca* triggered by changes in photoperiods.

### 4.4 Trade‐offs between reproduction and vegetative growth might optimize fitness under different conditions in the field

At the field site, the seeds of *C. chungii*, the host of *B. ochroleuca*, fall and germinate in November–December, when the average temperature is 15–20°C. The growing season starts in early spring with an average temperature of about 15°C and a relatively short photoperiod. During this period, the roots and collars of the seedlings are not totally lignified or suberized, and they are easy to infect. In addition, relatively suitable conditions are present for the mycelial growth of *B. ochroleuca*. Empirical evidence has emerged that suitable conditions for infection correspond to those for the vegetative growth of pathogenic fungi. For example, in tree infection caused by *Laccaria* *bicolor*, the optimal temperatures for fungal growth were similar to those for root lesion incidence, which represented infectivity, and lesion length, which represented mycelial growth (Strobel, 1991). It would be a beneficial strategy for *B. ochroleuca* to allocate more energy to growth rather than reproduction to fit the environment of moderate temperature and a short photoperiod. During the summer, the temperature increased (most often above 30°C between 13:00 and 15:00 on sunny days) and daytime lengthened. Most seedlings had suberized. Producing more spores would increase opportunities to encounter new habitats, that is, the tiny root tips that are suitable for infection and growth. Such a shift in life strategy from mycelial growth to sporulation has benefits for the fungal population. *B. ochroleuca* traded mycelial growth for more conidia under experimental conditions of a higher temperature (30°C) and a longer photoperiod. This plastic life‐history strategy of fungal pathogens implies a high adaptability to changing environments.

## CONFLICT OF INTEREST

The authors declare that they have no conflict of interest.

## AUTHOR CONTRIBUTIONS

**Yi Zheng:** Conceptualization (supporting); Data curation (equal); Formal analysis (equal); Resources (supporting); Software (equal); Writing‐original draft (equal). **Yichun Xie:** Data curation (equal); Formal analysis (equal); Methodology (equal); Writing‐original draft (equal). **Yan Xie:** Data curation (supporting). **Shixiao Yu:** Conceptualization (lead); Funding acquisition (lead); Project administration (lead); Resources (lead); Writing‐review & editing (lead).

## Supporting information

Supplementary Material

## Data Availability

The datasets generated during and/or analyzed during the current study are accessible in the Dryad Digital Repository (https://doi.org/10.5061/dryad.b2rbnzsfr).
