# Supplementary material for: Asexual reproduction and vegetative growth of Bionectria ochroleuca in response to temperature and photoperiod
Source: Ecol Evol. 2021 Jun 29;11(15):10515–25. doi: 10.1002/ece3.7856 (PMC8328416; doi:10.1002/ece3.7856)
Supplement: Supplementary file 1 — Supplementary Material [file ECE3-11-10515-s001.docx]

**Supplementary materials**

**The supplementary experiment to test the effects of inoculation concentration on mycelial growth and conidiation**

In order to confirm that inoculation concentration does not have strong influence on mycelial growth and conidiation, we inoculated the PDA plates with 0.5 μl spore suspension of two different concentrations (10^5^ μl^-1^ and 10^4^ μl^-1^) and incubated them in 25 ℃ with full-time light or darkness for 10 days. We compared colony sizes and the number of conidia (log transformed) between the groups of different inoculation concentrations, separately for the full-light group and full-darkness group, and vice versa. When incubated in full-time light, the colonies inoculated 0.5 μl of 10^5^ μl^-1^ suspension did not show significant different colony size (*t* = 1.39, *df* = 15.12, *P* = 0.19) or conidia production (*t* = 0.45, *df* = 5.67, *P* = 0.67) compared with those inoculated 0.5 μl of 10^4^ μl^-1^ suspension. When incubated in full-time darkness, the colonies did not show significant differences in sizes (*t* = -0.6194, *df* = 12.862, *P* = 0.5465) or conidia production (*t* = 1.43, *df* = 10.98, *P* = 0.18) between the groups with different inoculation concentrations, either. On the contrary, when inoculated with 0.5 μl of 10^5^ μl^-1^ suspension, colonies incubated in full-time light had significantly smaller colony sizes (*t* = -4.23, *df* = 14.30, *P* < 0.001) and higher conidia production (*t* = 2.75, *df* = 8.41, *P* = 0.02) compared to those incubated in full-time darkness. Similar trends were found when inoculated with 0.5 μl of 10^4^ μl^-1^ suspension (*t* = 6.97, *df* = 13.80, *P* < 0.001 for colony size; and *t* = 2.91, *df* = 6.53, *P* = 0.02 for conidia production). These results demonstrated that inoculation concentration had no strong effects on mycelial growth or conidia production of *Bionectria ochroleuca*.

**The blast result of Cch11:**

5’- **CCGTAGGTGAACCTGCGGAGGGATCATTACCGAGTTTACAACTCCCAAACC
CATGTGAACATACCTATTGTTGCTTCGGCGGGATTGCCCCGGGCGCCTCGTGTGCCCCGGATCAGGCGCCCGCCTAGGAACTTGAACTCTTGTTTTATTTTGAATCTTCTGAGTCGTTTTTACAAATAAATAAAAACTTTCAACAACGGATCTCTTGGTTCTGGCATCGATGAAGAACGCAGCGAAATGCGATAAGTAATGTGAATTGCAGAATTCAGTGAATCATCGAATCTTTGAACGCACATTGCGCCCGCCAGTATTCTGGCGGGCATGCCTGTCTGAGCGTCATTTCAACCCTCATGCCCCTAGGGCGTGGTGTTGGGGATCGGCCAAAGCCCGCGAGGGACGGCCGGCCCCTAAATCTAGTGGCGGACCCGTCGTGGCCTCCCCTGCGAAGTAGTGATATTCCGCATCGGAGAGCGACGAGCCCCTGCCGTTAAACCCCCAACTTTCCAAGGTTGACCTCAGATCAGGTAGGAATACCCGCTGAACTTAAGCATATCAATAAGCGGAGG -3’**

**Table S1. GenBank accessions of ITS sequences used in the phylogenetic analysis of *Bionectria ochroleuca* isolates in different lifestyles.**

**Fig. S1. The Neighbour-Joining phylogenetic tree built using ITS sequence of *Bionectria ochroleuca* isolates in different lifestyles.**

**Fig. S2. *Bionectria ochroleuca* colonies under high power microscope (400 ×).**

**Fig. S3. Conidia germination rate under different external nutrient supply.**

Table S1. GenBank accessions of ITS sequences used in the phylogenetic analysis of *Bionectria ochroleuca* isolates in different lifestyles.

| Sequence | Description | Lifestyle | Source | |
| --- | --- | --- | --- | --- |
| Cch11 | *Bionectria* *ochroleuca* isolate Cch 11 | Pathogen | *Cyclobalanopsis* *chungii* | |
| EU552110.1 | *Clonostachys* cf. *rosea* CBS 113336 culture CBS:113336 | Pathogen | *Protea* *nitida* | |
| FJ233188.1 | *Bionectria* sp. EXMQ-9 | Pathogen | Taiwan jujube | |
| HM849058.1 | *Bionectria* sp. UCD13Syrah | Pathogen | grapevine | |
| KP998525.1 | *Bionectria* sp. 2 MM-2016 | Pathogen | *Acacia mangium* | |
| KP998526.1 | *Bionectria* sp. 3 MM-2016 | Pathogen | *Acacia mangium* | |
| KP998527.1 | *Bionectria* sp. 4 MM-2016 | Pathogen | *Acacia mangium* | |
| KX421414.1 | *Clonostachys* *rosea* isolate AA2I32F2 | Pathogen | *Duponchelia fovealis* | |
| KY810797.1 | *Clonostachys* *rosea* isolate G1 | Pathogen | black-wattle minicutting | |
| KY810798.1 | *Clonostachys* *rosea* isolate G2 | Pathogen | black-wattle minicutting | |
| KY810806.1 | *Clonostachys* *rosea* isolate B2 | Pathogen | black-wattle minicutting | |
| MK713423.1 | *Clonostachys* *rosea* strain GKF21 | Pathogen | Fresh pepper fruit | |
| HQ731621.1 | *Bionectria* sp. Macof 02 | Endophyte | *Macleaya cordata* | |
| HQ731632.1 | *Bionectria* sp. Papochf 04 | Endophyte | Paris polyphylla Smith | |
| JF817324.1 | *Clonostachys* *rosea* strain CanS-48 | Endophyte | healthy canola stem | |
| JF817331.1 | *Clonostachys* *rosea* strain CanS-56 | Endophyte | healthy canola stem | |
| KM265525.1 | Fungal sp. E14424B | Endophyte | *Ocotea* *cuneifolia* | |
| Sequence | Description | Lifestyle | Source | |
| KX343152.1 | *Clonostachys* *rosea* isolate 22PDA-4C2 | Endophyte | mycorrhiza of Tuber melanosporum | |
| KX343160.1 | *Clonostachys* sp. isolate GETM11 | Endophyte | *Tuber melanosporum* ascocarp | |
| KY703873.1 | *Clonostachys* *rosea* strain pP3 | Endophyte | needles of Pinus ponderosa | |
| MT801140.1 | *Clonostachys* sp. isolate F3-1 | Endophyte | Strawberry | |
| MT801144.1 | *Clonostachys* sp. isolate F5-1 | Endophyte | Strawberry | |
| MH421858.1 | *Clonostachys* sp. strain MSX75296 | Endophyte/pathogen | terrestrial decaying wood | |
| HQ157202.1 | *Bionectria* *ochroleuca* isolate RZ | Free living | *Coprinus* *comatus* culture substrate | |
| KF367470.1 | *Bionectria* sp. 1 BRO-2013 | Free living | untreated drinking water sources | |
| KF367477.1 | *Bionectria* sp. 2 BRO-2013 | Free living | untreated drinking water sources | |
| KX058045.1 | *Clonostachys* *rosea* strain qlz6 | Free living | oil sample collected from apline grassland | |
| LC133846.1 | *Clonostachys* sp. JCM 28413 | Free living | Reddish substances on the stone of the south wall in the stone chamber of the Kitora Tumulus | |
| LC133855.1 | *Clonostachys* sp. JCM 28430 | Free living | Soil in burial mound of the Kitora Tumulus | |
| LT576164.1 | *Clonostachys* *rosea* | Free living | Truffle sediment | |
| MH911376.1 | *Clonostachys* *rosea* strain MF22417 | Free living | Loktak Lake, Manipur | |
| MN511326.1 | *Clonostachys* *rosea* isolate GS57 | Free living | *Lentinula* *edodes* culture substrate | |
| MT845995.1 | *Clonostachys* *rosea* isolate 14BCM | Free living | East Siberian Sea sediments | |
| MH681594.1 | *Clonostachys* sp. strain Bdf-4 | Symbiosis | *Blaptica* *dubia* | |
| MK120552.1 | *Clonostachys* *rosea* isolate 9 | Symbiosis | Digestive tract of *Phylloicus* *fenestratus* | |
| EU326186.1 | *Bionectria* *ochroleuca* isolate XSD-89 | Undetermined | *Huperzia* *whangshanensis* | |
| JF449849.1 | Uncultured *Bionectria* clone SW 2w A02 | Undetermined | beech litter | |
| Sequence | Description | Lifestyle | Source | |
| JF449861.1 | Uncultured *Bionectria* clone SW 2w B11 | Undetermined | beech litter | |
| JF449884.1 | Uncultured *Bionectria* clone SW 2w G03 | Undetermined | beech litter | |
| MH869057.1 | *Clonostachys* *rosea* strain CBS 376.55 | Undetermined | | Not available |
| MN889471.1 | *Clonostachys* *rosea* strain YS3-5-1 | Undetermined | | Not available |


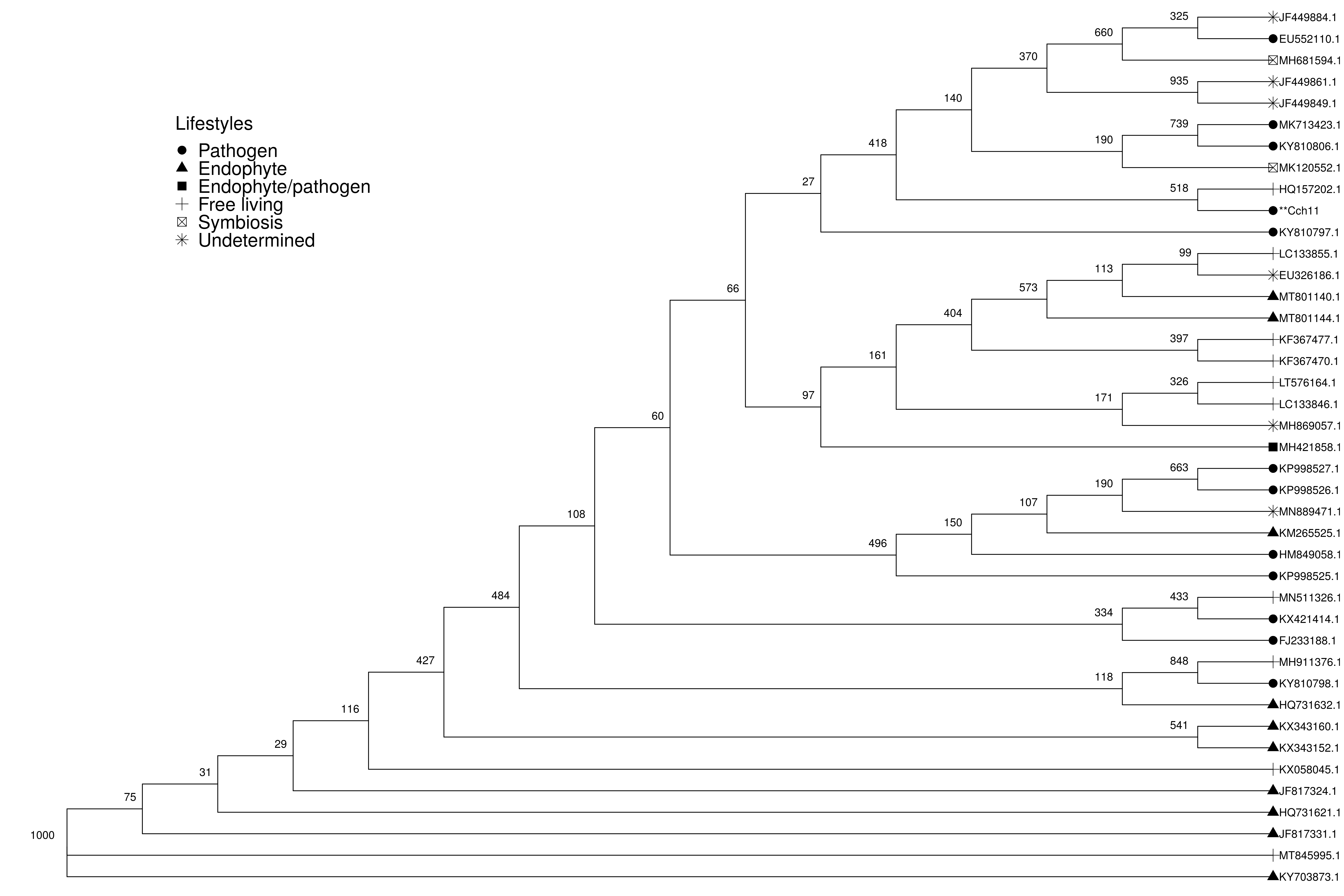


Fig. S1. The Neighbour-Joining phylogenetic tree built using ITS sequence of *Bionectria ochroleuca* isolates in different lifestyles. The evolutionary distances were computed using the neighbour joining method and Kimura's 2-parameters distance model. The bootstrap values inferred from 1000 pseudo replicates. The number of replicate trees in which the associated isolates clustered together in the bootstrap test are shown next to the branches.


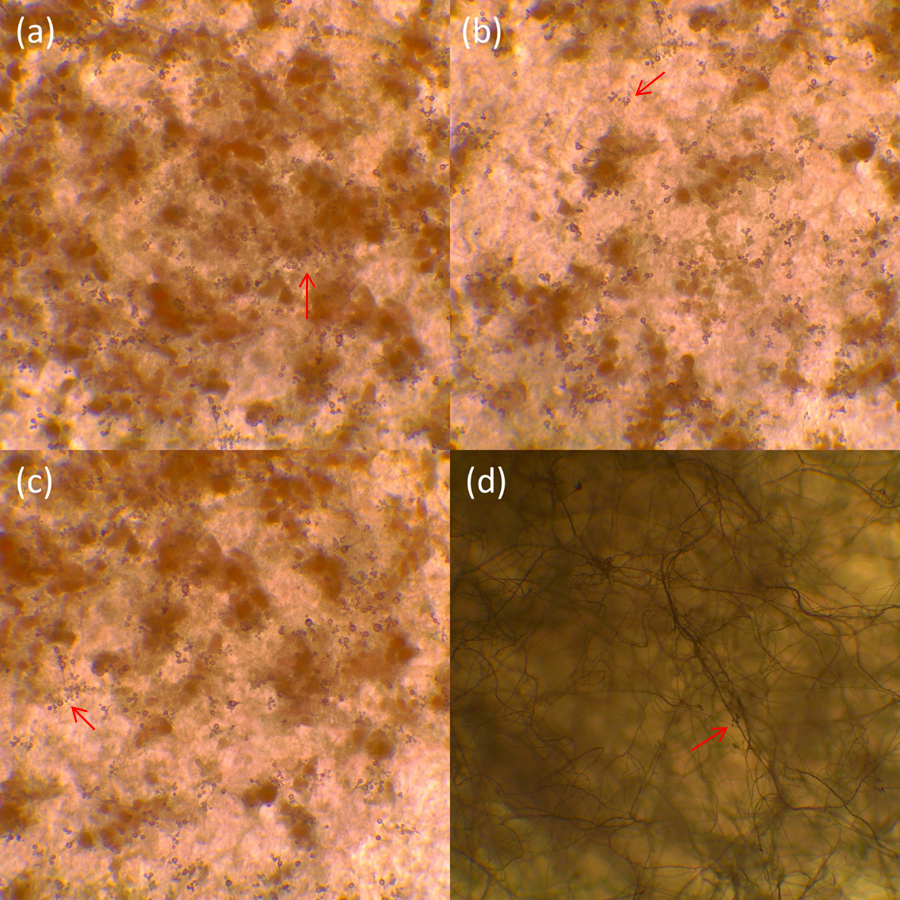


Fig. S2. *Bionectria ochroleuca* colonies under high power microscope (400 ×). (a) Dense area; (b) sparse area; and (c) intermediate area of the concentric ring. (d) Colony incubated under continuous darkness. Arrows point out the conidia.


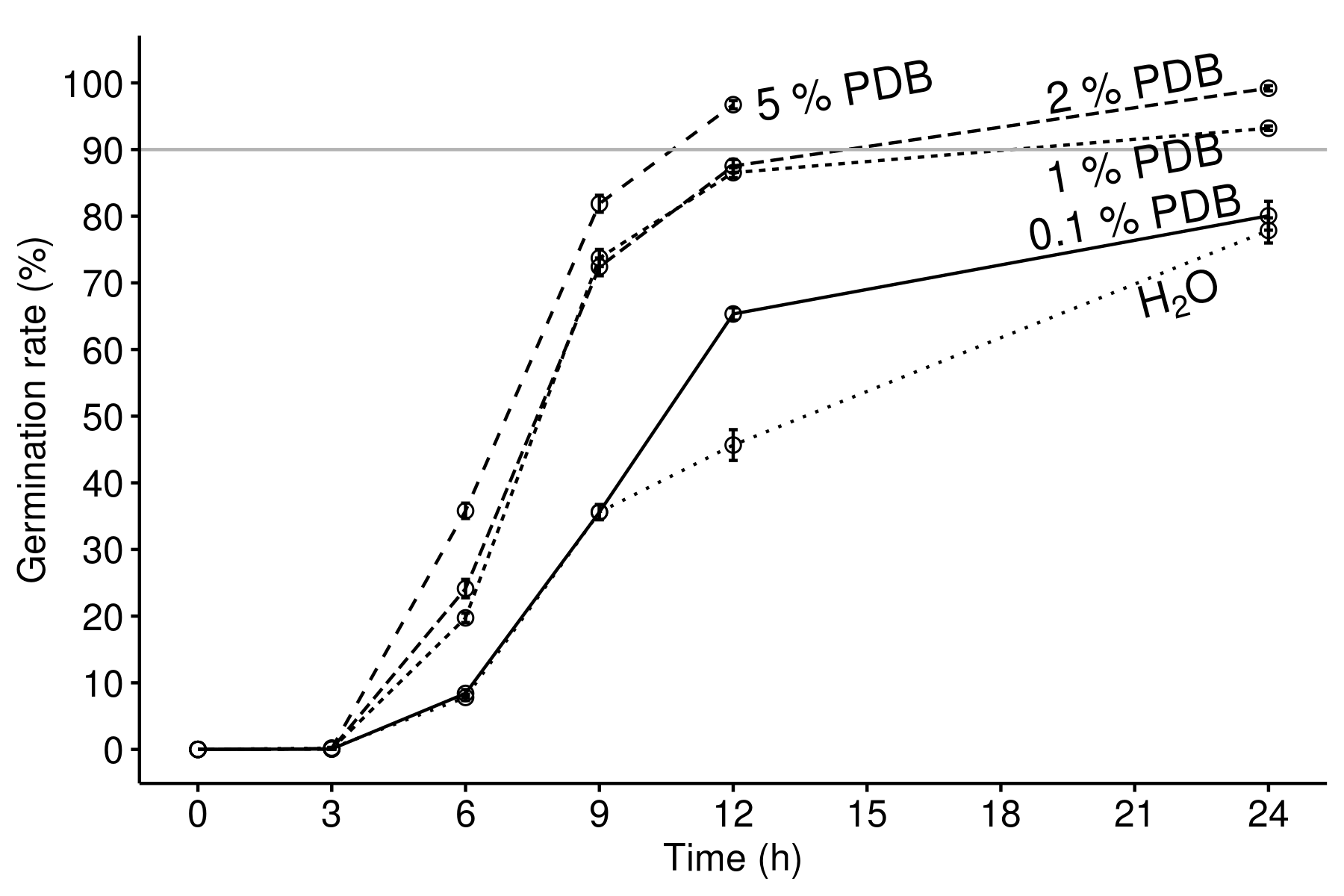


Fig. S3. Conidia germination rate under different external nutrient supply. Error bars show the standard error of 15 observation fields.
